# A rare complication of the hydatid cyst of the liver: A case report of Cysto-cutaneous fistula

**DOI:** 10.1016/j.ijscr.2025.112046

**Published:** 2025-10-10

**Authors:** Dorsaf Mouelhi, Ben lahouel Senda, Laila Jedidi, Mabrouk Aymen, Saidani Aya

**Affiliations:** a University of Tunis El Manar Faculty of Medicine of Tunis, Tunisia; b General Surgery Department, Regional Hospital of Jendouba, Tunisia; c Surgery A Department, Charles Nicolle Hospital, Tunisia

**Keywords:** Hydatid cyst, Liver, Cutaneous fistulization, Case report

## Abstract

**Introduction and importance:**

Hepatic Hydatid cyst of the liver is a parasitic zoonotic illness caused by Echinococcus granulosus that remains endemic in regions where sheep farming is common, particularly in our country, Tunisia. While it is a benign disease, its natural evolution can be marked by life-threatening complications. Cysto-biliary communication is the most common and serious complication. Spontaneous cutaneous fistulization is an extremely rare complication.

**Case presentation:**

We report the case of a 47-year-old female who presented with a chronic discharging sinus in the left upper quadrant of the abdomen. Clinical examination revealed a cutaneous opening with clear fluid discharge and surrounding inflammation. Abdominal imaging identified two hepatic hydatid cysts: one located in the left lobe, which was fistulized to the skin, and another in the right lobe. Surgical exploration confirmed the fistulous tract between the left hepatic cyst and the skin. A pericystectomy was performed on both cysts, along with excision of the fistulous tract.

**Clinical discussion:**

Cutaneous fistulization of a hepatic hydatid cyst is an infrequent complication, often resulting from chronic inflammation and gradual erosion of surrounding tissues. It may be misdiagnosed as a simple abscess, delaying appropriate treatment. Imaging techniques, particularly CT scans, are essential for diagnosis and surgical planning. Management relies on complete excision of the cyst and fistulous tract, combined with anti-parasitic therapy.

**Conclusion:**

Cutaneous fistulization of a hepatic hydatid cyst is a rare but serious complication that requires a high index of suspicion in endemic areas. Early imaging and prompt surgical intervention is essential to ensure complete recovery and prevent recurrence.

## Highlights

• Cutaneous fistulization is an uncommon serious complication of hydatid disease
• Establishing a preoperative diagnosis is particularly difficult in the absence of cystic material drainage from the external fistula tract.
• Management involves surgical intervention, such as cystectomy or hepatectomy with excision of the fistulous tract, in combination with medical therapy.
• Raising awareness is a key strategy in addressing hydatid disease and its complications.

The case report is reported in line with the SCARE criteria [1].

## 1 Introduction

Hydatid disease (HD) is caused by a parasite called Echinococcus granulosus. It is found in many parts of the world where sheep farming is common. These regions include the Mediterranean, the Middle East, South America, Central Asia, and sub-Saharan Africa. In North Africa, including Tunisia, the number of cases remains high, with reports ranging from 15 to 30 cases per 100,000 people each year. The liver is the most frequently affected organ [2]. While generally harmless, there is a risk of complications arising from various issues, particularly when it occurs in the bile duct [2]. A rare form of HD is when the disease affects the tissue under the skin, which then leads to the formation of skin fistulas. This phenomenon has only been observed in a limited number of cases, ranging from 0.1 % to 1.5 % [3]. In this paper, we present a rare case of a cysto-cutaneous fistula in a 47-year-old female patient treated in our department. This revealed a hydatid cyst in the left liver that had ruptured into the abdominal wall.

## 2 Case report

**Fig. 1:**
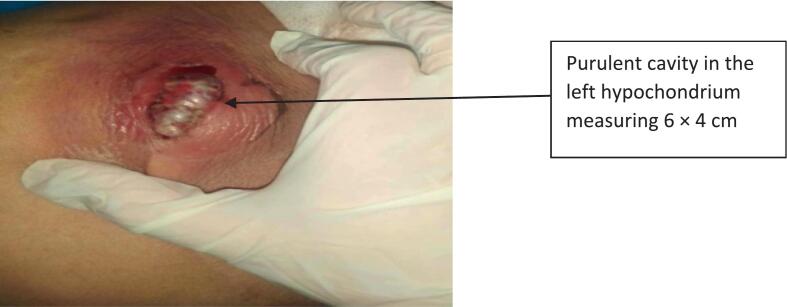
Subcutaneous abscess cavity.

**Fig. 2:**
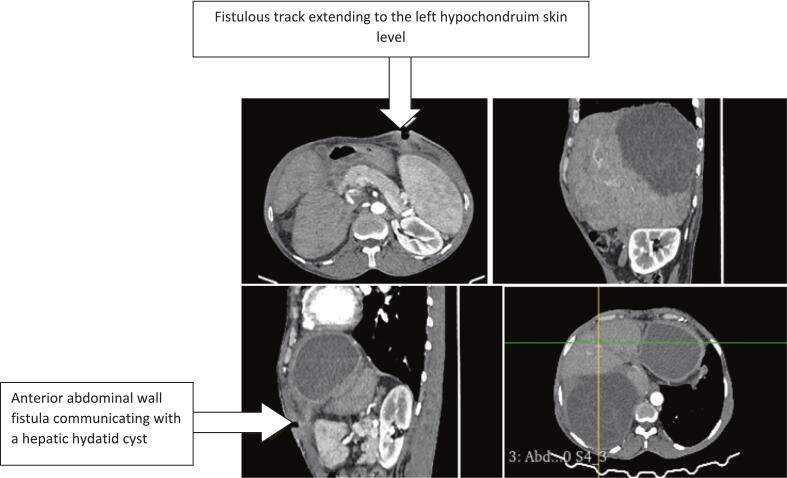
CT scan: subcutaneous rupture of a hydatid cyst of the left liver.

A 47-year-old female patient with no medical history underwent surgery for a hydatid cyst of the liver using a right subcostal incision fifteen years ago. She currently resides in a sheep-farming area and presented to our emergency department with upper right quadrant and epigastric pain, accompanied by vomiting, fever, and a cutaneous lesion with purulent discharge that appeared a week ago in the left hypochondrium (Fig. 1). During the physical examination, the patient was conscious but appeared generally unwell. She had a fever of 39 °C, but no signs of jaundice. The cardiovascular examination revealed no signs of tachycardia, with normal blood pressure (120/75 mmHg). The respiratory rate was found to be within normal parameters. A thorough abdominal examination revealed tenderness in the right hypochondrium. A tender mass was palpable in the left hypochondrium, and the examination of the lesion in the upper left quadrant revealed a fistula with purulent discharge. The rest of the examination revealed no further issues. Laboratory tests revealed an inflammatory biological syndrome characterized by elevated white blood cell counts (19,000 UI/ml) and C-reactive protein levels (153 mg/l), while liver function tests were found to be within normal parameters. An abdominal computed tomography (CT) scan revealed a 13-cm hydatid cyst in segments VII and VIII of the liver, along with a second, 7-cm multivesicular hydatid cyst in the left liver. The latter exhibited exophytic growth and had fistulized to the skin, accompanied by a heterogeneous subcutaneous fluid collection (Fig. 2). Due to limited access to advanced imaging modalities in our setting, as well as the absence of clinical or biological signs suggestive of cysto-biliary fistula, magnetic resonance cholangiopancreatography (MRCP) was not performed preoperatively. The diagnosis had already been established based on ultrasound and CT findings, which were considered sufficient for surgical planning. The hydatidosis serology was positive.

The patient was diagnosed with a liver hydatid cyst complicated by fistulization to the abdominal wall and a subcutaneous abscess. The patient received a 12 mg/kg/day dosage of albendazole for 15 days in conjunction with antibiotic treatment. Then we decided to operate on the patient. She was placed in the supine position with a slight reverse Trendelenburg tilt, the left and right sides elevated to allow optimal exposure of the left liver lobe. She then underwent surgery via a bi-subcostal incision. Two large hydatid cysts were found in the liver. The largest cyst, measuring 12 cm, was located in segments VII and VIII. The second cyst, located in the left lobe of the liver, exhibited a fistulous tract extending to the skin surface. Intraoperative indocyanine green (ICG) fluorescent imaging was not used, as this technology is not available in our center. The procedure involved an open cyst evacuation, followed by a partial pericystectomy of both cysts.

A cholecystectomy was performed by the surgical team for an intraoperative cholangiogram, and a trancystic drain was used. The results revealed no dilation of the common bile duct and no evidence of a cysto-biliary fistula. The residual cystic cavity was then cleared of its contents and irrigated with hypertonic (20 %) saline as a scolicidal agent. The fistulous tract was excised en bloc, and the cavity was externally drained. Three drains were placed in the hepatic and subcutaneous residual cavities. A postoperative cholangiogram on the fifth day showed no signs of bile leakage. The patient was discharged on the seventh day after the removal of the intra-cavitary drainage. Following the operation, she was prescribed a dosage of 10 mg/kg/d of Albendazole for six months. Postoperative events were assessed using the Clavien-Dindo classification system. In this case, the patient had an uneventful recovery, which corresponds to a Grade I complication. The procedure was performed by a surgeon with extensive experience in managing cases of hepatic hydatid disease in endemic regions, specifically in Jendouba, a city in the northwestern part of Tunisia. The patient expressed confidence in the surgical team and was pleased with the smooth postoperative recovery. She reported a high level of satisfaction with the care received and expressed gratitude for the successful outcome and resolution of her symptoms. Following a review at the six-month mark, the clinical evolution was found to be favorable. A follow-up abdominal CT scan performed at six months showed no residual or recurrent hepatic cysts or collections. Hydatid serology was repeated at six months and showed a significant decline in antibody titers, suggesting a good treatment response (Table 1).

**Table 1:** Clinical presentation.

Event	Details
Patient	47-year-old female
Surgery history	Previous hepatic hydatid cyst surgery 15 years earlier
Clinical presentation	Painful swelling with purulent cutaneous discharge in the left hypochondrium
Imaging	CT scan: two hepatic hydatid cysts; left-sided cyst fistulized to the skin
Treatment	Initial medical therapy, followed by: resection of cyst dome, fistula disconnection, and excision of fistulous tract
Postoperative course	Uneventful recovery; no complications or recurrence at 6-month follow-up

## 3 Discussion

Hydatid disease is a globally prevalent zoonotic infection caused by the larval form of the Echinococcus parasite. This issue continues to represent a notable public health concern in regions where the disease is endemic, with Tunisia being a prime example. While hydatid cysts can develop in any organ, the liver is by far the most frequently affected site. In most cases, hepatic hydatid cysts do not cause any symptoms; however, complications may result in the emergence of clinical symptoms. These may include secondary infection, anaphylactic reactions, compression of nearby structures, or rupture into the peritoneal cavity or biliary system. It should be noted that the most frequent complication is cyst rupture into the biliary tract [4]. In contrast, the extension of hepatic hydatid cysts into subcutaneous tissue with subsequent skin fistulization is extremely rare, occurring in only 0.1 % to 1.5 % of cases [3]. Following a thorough review of the literature on cutaneous involvement in hydatid disease (Table 2), we found 12 publications from 2015 to 2025. This type of complication is usually linked to cysts that are located in the front part of the body and exhibit exophytic growth patterns [5].

**Table 2:** Details of cases from review of literature for cutaneous fistulization of hydatid cyst of the liver.

Reference	Year	Country	Sex	Age	Cyst size (mm)	Cyst locations	Fistula opening/lesion location	Treatment
Algutaini & Al-Wageeh	2025	Yemen	Male	6	Not specified	Liver, abdominal wall	Epigastric region	Albendazole + surgical cystectomy
Ramraoui [9]	2024	Maroc	Not stated	68	87 × 69	Right liver lobe	Right hypochodruim	Surgery + albendazole
Mili [16]	2023	Tunisia	Male	10	50	Left liver lobe	Left hypochondrium	Albendazole + laparoscopic pericystectomy + tract drainage
Ramazan Orkun Önder [17]	2022	Turkey	Female	85	Not stated	Segments VI and VII of the liver	Not stated	A drainage catheter+medical treatment
Saif Ghabisha [10]	2021	Iran	Female	63	Not stated	Hepatic (unspecified lobe)	Abdominal wall	Laparotomy + partial cystectomy + tract excision
B. Khan [7]	2020	United Arab Emirates	Male	57	120 × 150 abscess mass	Multiple hepatic lobes	Right upper quadrant abdominal wall	Incision & drainage + albendazole
Cicero [12]	2017	Italy	Male	68	CE5 calcified cyst	Segment VI, right lobe	Right flank/subcutaneous abscess	Percutaneous drainage + surgical cystectomy + medical treatment
El Khoury [18]	2017	Lebanon	Female	68	55 × 80 × 56	Segments V–VIII, right hepatic lobe	Right breast	Albendazole + omentoplasty + cyst & tract drainage
Virgilio [19]	2015	Italy	Female	55	100	Right lobe liver	Right hypochodruim	Non operative management
Mandolkar [20]	2015	India	Female	25	110 × 100	Left lobe of liver	Left hypochondrium	Pericystectomy + fistula excision + albendazole
Daldoul [14]	2015	Tunisia	Female	23	50	Right lobe of liver	Right flank	Pericystectomy + fistula excision
Akay [21]	2015	Turkey	Male	80	100	Right liver lobe	Right hypochodruim	Drainage

This rare complication results from a combination of two main factors: mechanical and inflammatory processes [3]. Infection of the cyst contents can trigger inflammation of the pericyst, promoting its adherence and eventual fusion with the abdominal wall. Conversely, the thick and sometimes calcified pericyst gradually erodes the abdominal wall, a process that is further facilitated by repetitive respiratory movements.

Hydatid disease progresses through several stages before leading to an external rupture through the abdominal wall. In Stage I, the lesion extends to the innermost muscle layers. Stage II involves penetration beyond the muscular layer into the subcutaneous tissues. Stage III is characterized by the cyst reaching the subcutaneous layer and forming a cutaneous fistula [6].

Although uncommon, cutaneous fistulization is a serious complication of hydatid disease. A diagnosis can be challenging in cases where there is no visible discharge of cystic fluid or daughter vesicles from the fistula's external opening, particularly in regions where hydatid disease is endemic [7]. Clinical suspicion remains the cornerstone for early diagnosis and management. Key diagnostic indicators include residency in an endemic area and a known history of hydatid disease. From a clinical perspective, patients frequently present with a painful, febrile mass in the right hypochondrium. The presence of hydatid fluid or daughter cysts emerging from the fistulous tract is a valuable diagnostic sign [8,9]. A combination of clinical examination, imaging, and serological testing greatly aids in confirming the diagnosis [10]. Furthermore, histopathological analysis of any discharge from the fistula can confirm the parasitic origin and detect secondary infection, if present [6]. Abdominal computed tomography (CT) is a detailed diagnostic tool that can provide valuable insights into the nature of hydatid cysts and their associated fistulas. It is one of the most commonly used imaging methods for identifying cutaneous involvement and locating lesions responsible for complications associated with hydatid disease [11]. CT scans are particularly valuable in patients with a history of hydatid cyst surgery, as they help distinguish between a recurrent hepatic cyst causing a cysto-cutaneous fistula (as in the case we report) and a parietal inoculation of the abdominal wall during prior surgical intervention.

Positive hydatid serology is a valuable complementary tool to imaging, particularly in cases where the diagnosis remains uncertain. Of the available diagnostic techniques, contrast-enhanced fistulography is particularly useful in evaluating cutaneous complications of hydatid disease. It provides precise information regarding the extent of the fistulous tract, the location and size of the involved lesion, and its anatomical relationship with the biliary tree [6].

It is essential that the presence of a cysto-biliary fistula is identified before the procedure, and this should be done using magnetic resonance cholangiography as a standard procedure [12]. When necessary, intra-operative cholangiography can be used as a complementary method to further delineate the biliary anatomy and confirm the presence of a fistulous connection [13].

Cutaneous fistulization is generally not sufficient to achieve complete healing, as it only permits partial drainage of the abscess and the hydatid cyst [14]. Therefore, surgical intervention remains necessary to ensure the complete elimination of all parasitic fragments. A 4-week course of Albendazole should be taken before surgery. The combination of surgical and anti-parasitic treatment has shown favorable outcomes in patients suitable for operative management [14,15]. The surgical approach typically involves cysto-parietal disconnection and the removal of any secondary abscess, followed by definitive treatment of the hydatid cyst, management of the residual cavity, and excision of the fistula tract. Albendazole therapy is strongly recommended to reduce the risk of recurrence [13].

In cases of chronic cutaneous discharge in the right or left upper quadrant, particularly in endemic areas, several differential diagnoses should be considered. These include pyogenic liver abscesses with spontaneous cutaneous drainage, tuberculous liver abscesses, infected hematomas, necrotic liver tumors, particularly when imaging is nonspecific or serology is inconclusive, thus delaying appropriate management. In high-risk cases of cutaneous fistulization due to hydatid disease, especially in endemic regions and among patients with a history of hepatic hydatid disease, early diagnosis and prevention of potentially dangerous interventions such as blind drainage or incomplete cyst excision are essential.

## 4 Conclusion

Hydatid disease is a parasitic infection caused by the parasite Echinococcus granulosus. While it is generally considered to be a harmless condition, it can potentially lead to more severe outcomes due to associated complications. Spontaneous cysto-cutaneous fistulization of the hydatid cyst is an exceptional complication of hydatid disease. Imaging plays a key role in facilitating positive diagnoses and effective postoperative monitoring. Surgical treatment involving the excision of the cyst and fistula tract, in combination with medical intervention, has proven to be an effective approach for the management of hydatid cysts that result in cutaneous fistulas.

## CRediT authorship contribution statement

Mouelhi Dorsaf: Data collection, manuscript writing.
Laila Jedidi; Senda ben Lahouel and Aymen Mabrouk: Critical supervision, revising manuscript.
Aya Saidani: Resources, visualization, redaction.

All authors agreed to the final manuscript.

## Patient consent

Written informed consent was obtained from the patient for publication of this case report and accompanying images. A copy of the written consent is available for review by the Editor-in-Chief of this journal on request.

## Ethical approval

Ethical approval is exempt/waived at our institution.

## Guarantor

Mouelhi Dorsaf.

## Research registration number

Not applicable.

## Provenance and peer review

Not commissioned, externally peer reviewed.

## Funding

The study was not supported by any sponsor or funder.

## Declaration of competing interest

The authors declare that they have no known competing financial interests or personal relationships that could have appeared to influence the work reported in this paper.
